# Absence of Hepatitis B Resistance Mutants before Introduction of Oral Antiviral Therapy

**DOI:** 10.1155/2013/130384

**Published:** 2013-09-12

**Authors:** Martin Moehlen, Maria De Medina, Mary Hill, Lennox Jeffers, Eugene R. Schiff, Paul Martin

**Affiliations:** ^1^Section of Gastroenterology & Hepatology, Department of Internal Medicine, School of Medicine, Tulane University, 1430 Tulane Avenue, SL-35, New Orleans, LA 70112, USA; ^2^Center for Liver Diseases, University of Miami, School of Medicine, 1500 NW 12th Avenue, Suite 1101, Miami, FL 33136, USA

## Abstract

*Introduction*. The aim of this study was to assess whether hepatitis B virus drug resistant mutations antedated the widespread use of nucleos(t)ide analogues in treatment naïve patients. A number of reports have suggested that drug resistant mutants can be detected in apparently treatment naïve patients. *Study*. Fifty deidentified serum samples collected from 1986 to 1992 from patients with replicative chronic HBV infection at the University of Miami were genotyped and tested for resistance mutations using a line probe assay InnoLiPA HBV DR v2/v3. Serum HBV DNA was measured. All patients had documented chronic HBV infection with a detectable viral load, HBeAg seropositivity, and absence of HIV infection. *Results*. Of the 50 individuals included, 86% were male, mean age was 40 ± 12 years, and mostly genotype A. The mean HBV DNA was 126 pg/mL (range 6.4 to 557.0). No mutations were identified. *Conclusions.* The absence of drug induced mutations in these sera collected several years prior to the introduction of oral antiviral therapy suggests that these mutations do not occur in treatment naïve populations. Detection of drug resistance in an apparently treatment naïve subject suggests either unrecognized prior antiviral therapy or infection by an inoculum from a treatment experienced patient.

## 1. Introduction

Infection with hepatitis B virus (HBV) remains a frequent etiology of cirrhosis and hepatocellular carcinoma with estimated 360 million individuals with chronic infection worldwide [1]. Although vaccination will ultimately reduce the disease burden due to HBV, antiviral therapy can favorably alter its natural history [2]. Goals of therapy include suppression of HBV replication to delay progression of liver disease reducing the risk of hepatic decompensation and hepatocellular carcinoma as well as infectivity to other individuals. Although standard interferon alpha was licensed for the treatment of chronic HBV infection over two decades ago more widespread therapy became feasible with the advent of well-tolerated oral antiviral nucleoside and nucleotide agents. However, shortly after the introduction of the first nucleoside analogue, lamivudine, treatment induced mutations in the viral genome were identified. These mutations resulted in reduced antiviral efficacy and diminished clinical benefit. Subsequently adefovir, a nucleotide analogue, efficacious in presence of lamivudine induced mutations was licensed, but its use was also associated with emergence of genomic antiviral resistance. Antiviral resistance has also been identified with more recently introduced nucleoside analogue, telbivudine, and entecavir but to date no clinically significant mutations have been recognized with tenofovir, a nucleotide analogue.

Society guidelines and treatment algorithms endorse close monitoring of subjects treated with oral antiviral therapies for development of viral mutants [3–5]. A resistance mutation is an amino acid change that decreases sensitivity to an antiviral drug. Mutations can be divided into primary and secondary mutations, the former an amino acid substitution that results in decreased susceptibility to an antiviral agent, whereas the latter is compensatory amino acid substitution(s) restoring the functional defect in the HBV polymerase activity associated with primary drug resistance; hence, secondary mutations restore replicative fitness to HBV [5]. The primary resistance mutations are rtM204V/I within the YMDD motif for lamivudine, rtN236T, and/or rtA181T/V for adefovir. Telbivudine has a high degree of cross-resistance with lamivudine, with its primary resistance mutation M204I and secondary mutations L80I/V or L180M [6]. Entecavir has two distinct patterns of resistance that both include the YMDD mutation associated with lamivudine resistance, rtI169T + rtL180M + rtM204V + rtM250V and rtL180M + rtT184G + rtS202I + rtM204V; however, other resistance patterns have been reported [5]. 

HBV replication includes an inherently error prone reverse transcriptase, as it does not have a 3′-5′ proofreading exonuclease activity. On average approximately 10^11^ virions are produced daily [25], with estimated 8 × 10^−5^ mutations/base pair/year [26]. Exposure to oral antiviral agents selects naturally occurring mutations with intrinsic antiviral resistance. Generally these less replicatively fit than “wild type” HBV and therefore do not persist in circulating blood in the absence of an agent inducing resistance. Although drug resistant mutants are selected by antiviral therapy, a number of reports have described their identification in apparently treatment naïve subjects with lamivudine resistance mutations, specifically the M204I/V ranging from 0.8 to 27% [7, 9, 10, 13, 15, 17, 18, 21, 22]. Implications of HBV resistance mutants in treatment naïve patients include concerns about the inappropriate use of agents with similar resistance patterns. Furthermore transmission of HBV resistant mutants to others has been recognized. Generally reports of detection of antiviral resistant mutants in treatment naïve patients have been in sera collected after the introduction of the oral antiviral agents; further, several reports do not specify the timeframe from which sera was collected hence making interpretation of this relationship more difficult. The aim of our study was to determine whether resistant mutations were present before the era of antiviral HBV therapy. 

## 2. Methods and Materials

We used deidentified residual blood sera collected from July 1986 to July 1992 at the University of Miami. Blood samples were centrifuged, aliquoted, and then stored at −20°C until testing. The study was approved by the University of Miami Institutional Review Board. All of the patients had chronic HBV infection (detectable HBsAg positive for at least 6 months) with a detectable viral load (at least 10 pg/mL), HBeAg seropositivity, and absence of HIV coinfection (negative anti-HIV by enzyme immunoassay). All sera were obtained from patients naïve to oral antiviral therapy. Serological markers of HBV were analyzed using commercially available enzyme immunoassay kits (Abbott Laboratories, Abbott Park, IL and Roche). HBV DNA assay was performed with the Abbott Liquid Hybridization HBV DNA quantitative assay (Genostics, Abbott, Chicago, IL). Genotyping was done with the INNO-LiPA HBV genotype kit (Innogenetics, Belgium). Amplification of HBV DNA was completed with the Roche high pure viral nucleic acid kit (Roche Diagnostics, Indianapolis, IN) and the mutation analysis was accomplished with the INNO-LiPA HBV DR v2/v3 (Innogenetics, Belgium). The INNO-LiPA HBV DR v2/3 reverse hybridization assay can detect single nucleotide mismatches associated with lamivudine, adefovir, and entecavir resistance comprising 5% or more of the viral population [5]. Descriptive statistics were processed using the SAS 9.2 program package (SAS Institute, Inc., Cary, NC). 

## 3. Results

Of the 50 patients whose sera were included 86% were male, average age was 40 ± 12 years, 86% were genotype A, 6% were genotype F, 3.5% were genotype D, and 3.5% were genotype C with a mean HBV DNA of 126 pg/mL (Table 1). No detectable mutations were present in 47 of the 50 samples with INNO-LiPA HBV DR 2/3 testing. Three of the 50 samples had indeterminate results for certain codons (180/181, 236, and 250); these three samples were submitted to InnoGenetics Laboratories in Belgium where direct PCR sequencing confirmed that there were no mutations within these codons. 

## 4. Discussion

Our results suggest that prior to the introduction of oral antiviral agents HBV resistant mutants were absent in treatment naïve patients. Detection of HBV resistant mutants in treatment naïve patients in other reports has a number of possible explanations. Patients may have contracted HBV by contact with a treatment experienced patient. Alternatively patients may have had unrecognized exposure to antiviral agents, for instance, contained in herbal preparations. A number of other groups have reported the presence of resistant HBV mutants in treatment naïve patients. To determine whether these reports were pertinent the following criteria were applied: (1) treatment naïve hepatitis B treatment, (2) absence of HIV coinfection, (3) hepatitis B DNA polymerase mutation testing performed (any method), (4) the time period of blood sera collection specified, and (5) including 10 or more subjects. We identified 18 reports, including abstracts, for further analysis (Table 2). Only one of the eighteen papers examined sera in the before 1995 era, Matsuda et al. [7]. An additional paper performed a phylogenetic cluster analysis on HBV sequences before 1995 from the National Center Biotechnology Institute database [13]. 

Matsuda et al. evaluated 50 Japanese patients' sera, the majority obtained pre-1995. Twenty (40%) were asymptomatic carriers, and sera were assessed by PCR (both by enzyme-linked minisequence assay and by restriction fragment length polymorphism). Sera were collected from 1979 to 1989 and the YMDD motif was specifically sought. No mutations were noted. A second group of 10 (20%) individuals had their sera examined prior to lamivudine therapy and were assessed for surface antigen loss. All screening sera were collected from 1976 to 1992 and sequenced by PCR for YMDD mutation and found to be negative. Lastly, there was a third group of 20 individuals who were treated with lamivudine; however, prior to initiating lamivudine therapy, the YMDD motif was assessed among treatment subjects, and sera had been collected from 1994 to 2000. No treatment induced mutations were detected in this group [7].

In another report Chinese investigators sought to determine if widespread oral antiviral use had increased resistance to therapy and whether drug resistant strains had been transmitted by patients [13]. Direct PCR sequencing was performed from 328 treatment naïve patients, predominantly genotype C and HBeAg positive. Approximately 4% of patients had lamivudine associated viral resistance mutations, less than 1% had adefovir (0.6%), and entecavir (0.3%) related viral mutants. Phylogenetics were performed to assess the genetic distance between HBV reverse transcriptase sequences in sera collected between 2005 and 2006 and controls submitted to the National Center for Biotechnology Information (GenBank) before 1995. Phylogenetic analysis is a method used to assess genetic evolutionary changes over a period of time. The cohort compared to the genetic bank was found to be similar (genetic distance of 0.02), allowing the authors to conclude that the advent of nucleos(t)ide therapy did not accelerate evolutionary change [13]. 

 Kobayashi et al. [9] reported the presence of viral mutants in eighteen treatment naïve chronic HBV patients. 13 (72%) of whom had the YMDD wild type, while 5 (28%) had mutants. Samples had been collected between 1997 and 1999 from treatment naïve patients. Other reports, with the exception of Matsuda et al. [7], (Table 2) had samples from 1995 onwards, all with variable results and frequencies of viral mutants. 

There are some limitations to our study. First, the sample size is relatively small. From our literature search, of the eighteen selected papers (Table 2), 75% had sample sizes larger than 50, with the average size including approximately 170 patients. Second, we had three of the fifty samples that had indeterminate results on the INNO-LiPA DR2/3 testing. These samples were confirmed to not have resistance mutations; however, at the expense of a relatively less sensitive test, direct PCR sequencing. Third, reverse hybridization assay, such as INNO-LiPA is known to be extremely sensitive (able to detect viral resistance constituting 5% or more of the total viral population); however, the test does not detect novel mutations and may not be as sensitive as ultradeep pyrosequencing (UDPS) [10, 27]. Finally as the samples were deidentified limited demographic information could be obtained about the subjects, such as ethnicity. In regard to ethnicity, however, as the majority of subjects had genotype A, one could infer that they were of North American-European descent.

In conclusion, our results support the hypothesis that HBV drug resistance mutations did not antedate introduction of oral antiviral agents and that the detection of drug resistance in an apparently treatment naïve subject suggests either unrecognized prior antiviral therapy or infection by an inoculum from a treatment experienced patient.

## Figures and Tables

**Table 1:** Clinical and laboratory characteristics of the study population.

Characteristic	Values
Patients *N*	50
Male *N* (%)	43 (86)
Female *N* (%)	7 (14)
Years of age, mean ± SD (range)	40 ± 12 (19–65)
HBV DNA, mean pg/mL ± SD (range)	126 ± 121 (6–557)
HBV genotype *N* (%)	
A	43 (86)
B	0
C	2 (3.5)
D	2 (3.5)
E	0
F	3 (6)

**Table 2:** Presence of DNA polymerase mutations in chronic monoinfected hepatitis B treatment naïve.

Author, year	Country	Time frame	*N*	Method(s)	Presence of DR mutations	Presence of LAM DR mutations
Matsuda et al. 2004 [7]	Japan	1976–2000	50	PCR	N	N
Matsuda et al. 2004 [8]	Japan	1995–2001	71	PCR	Y	Y
Kobayashi et al. 2001 [9]	Japan	1997–1999	18	PCR	Y	Y
Margeridon-Thermet et al. 2009 [10]	United States	1998–2007	17	PCR/UDPS	Y	Y
Amini-Bavil-Olyaee et al. 2008 [11]	Iran	2002–2006	147	PCR	N	N
Pollicino et al. 2009 [12]	Italy	2002–2007	100	PCR	N	N
Han et al. 2009 [13]	China	2005-2006	328	PCR	Y	Y
Nguyen et al. 2009 [14]	United States	2005–2008	472	PCR	Y	Y
Fung et al. 2008 [15]	Canada	2006–2008	209	RHA	Y	Y
Liu et al. 2010 [16]	China	2007-2008	192	PCR	N	N
Mirandola et al. 2011 [17]	Italy	2007–2009	255	RHA	Y	Y
Fung et al. 2009 [18]	Canada	2007–2009	311	RHA	Y	Y
Salpini et al. 2011 [19]	Europe	2007–2010	140	PCR	Y	N
Sayan et al. 2010 [20]	Turkey	2008-2009	88	PCR	Y	N
Osiowy et al. 2011 [21]	Canada	2008-2009	201	RHA/UDPS	Y	Y
Ng et al. 2009 [22]	Canada	2008-2009	175	RHA	Y	Y
Nguyen et al. 2009 [23]	United States	2009	70	RHA	N	N
Nguyen et al. 2011 [24]	United States	2009–2011	200	RHA	Y	N

Key: time frame: time range of blood sera collection; *N*: number of naïve treated subjects; method(s): method(s) used to detect genotypic resistance mutations; DR: drug resistance; LAM: lamivudine; PCR: polymerase chain reaction; UDPS: ultradeep pyrosequencing; RHA: reverse hybridization assay; Y: yes; N: no.
